# Developing Tools to Counteract and Prevent Suicide Bomber Incidents: A Case Study in Value Sensitive Design

**DOI:** 10.1007/s11948-016-9832-8

**Published:** 2016-11-28

**Authors:** Lambèr Royakkers, Marc Steen

**Affiliations:** 1 Eindhoven University of Technology, P.O. Box 513, 5600 MB Eindhoven, The Netherlands; 2 Netherlands Organisation for Applied Scientific Research (TNO), Anna van Buerenplein 1, 2595 DA The Hague, The Netherlands

**Keywords:** Value Sensitive Design, Ethics, Terrorism, Suicide bomber

## Abstract

Developers and designers make all sorts of moral decisions throughout an innovation project. In this article, we describe how teams of developers and designers engaged with ethics in the early phases of innovation based on case studies in the SUBCOP project (SUBCOP stands for ‘SUicide Bomber COunteraction and Prevention’). For that purpose, Value Sensitive Design (VSD) will be used as a reference. Specifically, we focus on the following two research questions: How can researchers/developers learn about users’ perspectives and values during the innovation process? and How can researchers/developers take into account these values, and related design criteria, in their decision-making during the innovation process? Based on a case study of several innovation processes in this project, we conclude the researchers/developers involved are able to do something similar to VSD (without them knowing about VSD or calling it ‘VSD’), supported by relatively simple exercises in the project, e.g., meetings with potential end-users and discussions with members of the Ethical Advisory Board of the project. Furthermore, we also found—possibly somewhat counterintuitively—that a commercial, with its focus on understanding and satisfying customers’ needs, can promote VSD.

## Introduction

Developers and designers make all sorts of moral decisions throughout an innovation project. Many of these decisions concern values such as health, safety, efficiency, and privacy. Often, developers and designers use these values as choice criteria, but sometimes values conflict and moral decisions need to be made (Steen 2015; Van den Hoven et al. 2015). Below, we describe how teams of developers and designers engaged with ethics in the early phases of innovation. Our descriptions are based on case studies in the SUBCOP project (SUBCOP stands for ‘SUicide Bomber COunteraction and Prevention’). This project was part of the 7th Framework Programme for Research and Technological Development and ran from June 2013 to July 2016. SUBCOP was concerned with the study and development of tools, technologies or procedures that can be used during incidents with a threat of a suicide bomber attack. More specifically, the project aimed to develop ‘less than lethal’ tools and technologies that can help to counteract and prevent suicide threats with a ‘Person Borne-Improvised Explosive Device’ (PB-IED). The deployment of such less-than-lethal weapons has ethical implications. Although the goal of such weapons is to minimize violence, the effects can be the opposite, e.g., in the case of tasers used by the police. These were originally intended for cases that require an intervention more harmful than shouting but less harmful than shooting. In practice, however, tasers are sometimes excessively used or even abused (Amnesty International 2004).

Regarding ethics, we can distinguish two main ‘moments’ in which ethical decision are made:

1. During the project, when project team members make decisions, in research and design activities, e.g., when they select specific less-than-lethal tools, or when they articulate specific tactical procedures.
2. During a suicide bombing threat, e.g., when police officers interact with a suicide bombing suspect; they will then choose between different tools or interpret the procedures that come with these tools.

In this paper we will focus on the former, i.e., on ethical decision-making in the SUBCOP project, and we will use Value Sensitive Design (VSD) as a reference. VSD was put forward by Batya Friedman and others as an approach to proactively consider moral values during the process of technological design (Friedman and Kahn 2003; Friedman et al. 2006). In VSD it is assumed that these values become embedded in the technologies that are developed, and that these values shape the space of action of users; they offer opportunities and constraints to the people who interact with these technologies. VSD advocates taking into account different values of different stakeholders throughout the process of research, development and design. VSD can be thought of as a form of Responsible Research and Innovation in that it aims to organize a “transparent, interactive process by which societal actors and innovators become mutually responsive to each other with a view to the (ethical) acceptability, sustainability and societal desirability of the innovation process and its marketable products” (Von Schomberg 2011, 9).

The practice of VSD is characterized by a diversity of approaches, theoretical backgrounds, values for which it is designed, and application domains (Van den Hoven et al. 2015). To date, however, VSD has been primarily applied in information systems design and human–computer interaction. The objective of this paper is to extend this application, by looking into the development of non-lethal weapons and tools; a domain that involves questions about life or death—questions that are, in a sense ‘more serious’ than questions about, e.g., privacy that one will commonly find in information systems design and human–computer interaction. Furthermore, our objective is to study practices of researchers and developers that engage in something-like-VSD without them knowing about VSD or calling it ‘VSD’. This would be supplementary to existent studies of VSD, which typically describe projects that involve people from academia that specialize in VSD. We hope that our findings will be useful for projects which have aims similar to VSD, but which do not involve experts of VSD.

Below, we will proceed in the following way. First we briefly discuss VSD and some of its critiques. Then we introduce the SUBCOP project and our case study approach. Then we provide descriptions of five research and development processes, focusing on how researchers and developers took into account moral values and considerations. Then we present a discussion, conclusions and recommendations for practitioners.

## Value Sensitive Design

Value Sensitive Design evaluates and informs the development of technologies by taking into account moral values. It sets out an integrative and iterative tripartite methodology, juggling and keeping in play the results of conceptual, empirical, and technological investigations (Van de Poel and Royakkers 2011):

1. Conceptual investigations aim at identifying the stakeholders and clarifying the values at stake, and at making trade-offs between the various values. We distinguish direct (those who use or will use the technology or method) and indirect stakeholders (those who may not engage the technology or method directly, but whose lives will be influenced through the use of the technology or method). In the SUBCOP project, a conceptual investigation of an on-site surveillance installation identifies the security personnel who can monitor the scene and redirect crowds or close down specific areas as direct stakeholders. The indirect stakeholders include the public whose images will be captured by the cameras.
2. Empirical investigations aim at understanding the contexts and experiences of the people affected by technological designs/methods. Such investigations may employ a variety of methods: surveys, questionnaires, interviews, experiments, participant observations, etc. This is relevant to appreciating precisely what values are at stake and how these values are affected by different designs.
3. Technical investigations analyse designs/methods and their operational principles to assess how well they support particular values, and, conversely, to develop new innovative designs/methods that meet particular morally relevant values particularly well.

This methodology provides the opportunity to develop new technical or procedural options that more adequately meet the values of ethical importance than current options do. VSD, however, has received criticism for a tendency to privilege the researchers’ own perspectives or values over the perspectives or values of stakeholders. This happens, e.g., when researchers imagine stakeholders and their perspectives and values, rather than enabling these stakeholders to actually express their perspectives themselves and to participate in the innovation process. According to Yetim (2011), VSD fails to provide a systematic and comprehensive method for identifying stakeholders, and fails to address the use of deliberative methods and tools to promote joint reflection on values during the design process—in particular, reflection by stakeholders on their own values, value tensions, and implications for design, as participants in the design process. Others, too, have called for greater stakeholder participation in the VSD process (Kujala and Väänänen-Vainio-Mattila 2008; Pommeranz et al. 2011).

Borning and Muller (2012) further argue that stakeholders should have a greater voice in the VSD process, and raise concerns how the multiplicity of voices is identified, brought forward, and attended to throughout the lifecycle of a project. They recommend that VSD scholars consider stakeholder participation and voice throughout the entire research process. This could be established by incorporating practices of Participatory Design (Schuler and Namioka 1993; Iverson et al. 2010; Halloran et al. 2009), in the VSD process. The methodology “is based on the genuine decision-making power of the do-designers and the incorporation of their values in the design process and its outcome, which is often a high-fidelity prototype for a product or service, or a new way to organize a work practice or to design a space” (Van der Velden and Mörtberg 2015, 42).

## Case Study: SUBCOP

The SUBCOP project “addresses the extraordinary challenge of how to intervene in a [suspected] suicide bombing event using less-than-lethal means” (http://www.subcop.eu/). Its core objectives were: (1) to consider available technological tools for less-than-lethal intervention; (2) to consider novel procedures for their application; and (3) to develop new less-than-lethal capabilities, e.g., tools or procedures.

The project proposal featured a figure that illustrates various possible, novel tools and procedures:
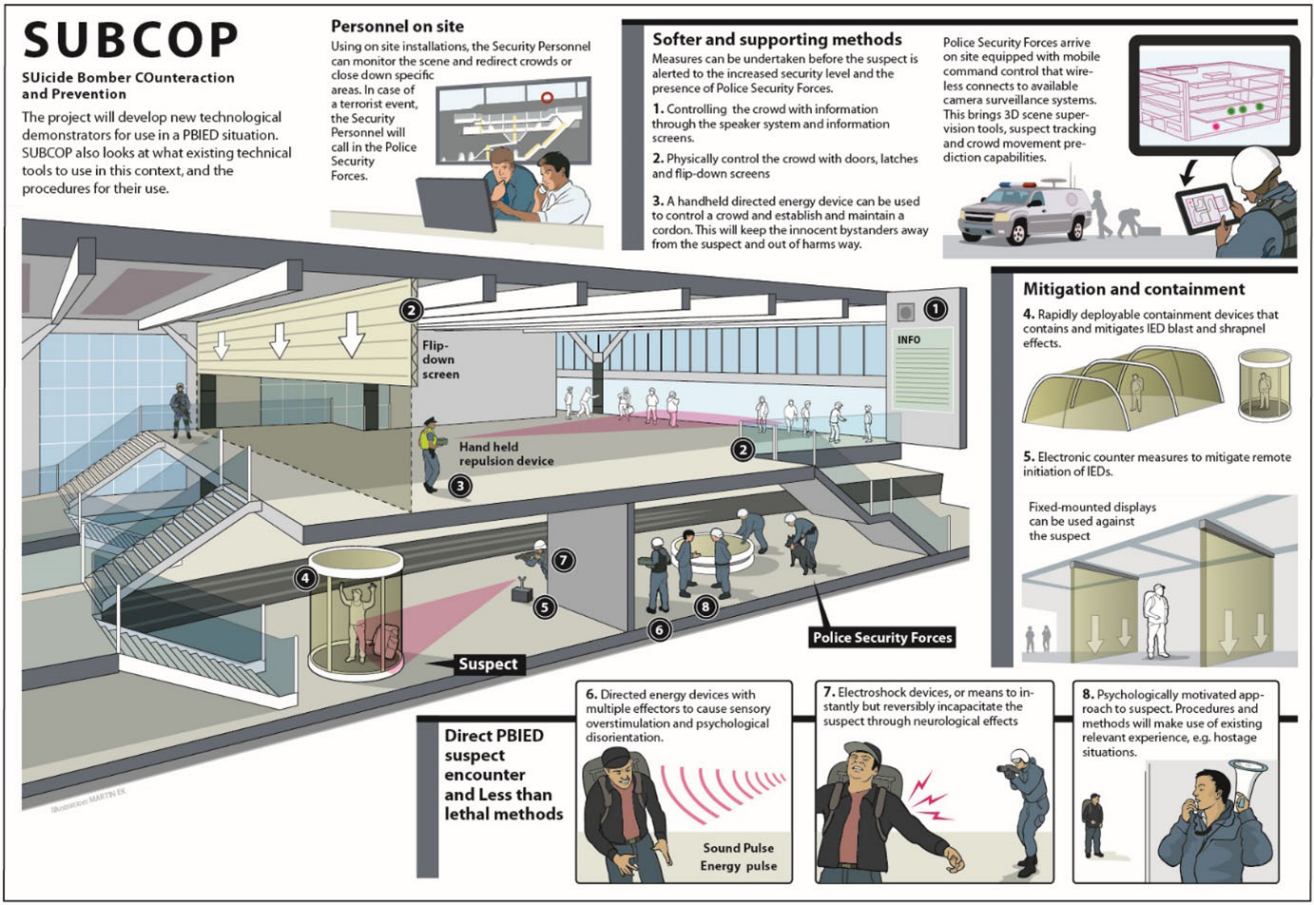


The project paid significant attention to end-users, in at least two distinctive ways: (1) two public security agencies were involved in the project as partners (Ministry of Public Security of Israel and Police Organization of Turkey); and (2) multiple police organizations (from Austria, France, Germany, The Netherlands, Israel, Sweden and the United Kingdom) were involved in a series of End-user Meetings (every 6 months). The objectives of these end-user involvement efforts were the following:

1. To make the Suicide Bomber Counteraction and Prevention programme applicable to the end-users who will make the operational decisions;
2. Identify the problems faced by the end-user when managing a suicide bombing incident;
3. To identify the operational scenarios likely to be faced during a suicide bombing incident;
4. To set up an End-user Advisory Board and End-user Platform.

Although the project did not organize an explicit VSD process, efforts were made that are comparable to VSD: one type of stakeholder was focused on, namely end-users; police officers were involved as active and creative participants, and their interests and values were studied in detail and were taken into account in many research and development efforts.

Moreover, these police officers were also able to express interests and values of other stakeholders, in particular of by-standers (in the incident) and of suicide bombing suspects (both in cases in which they prove to be suicide bombers, and in cases where they prove to be innocent). So the police officers acted as spokespersons for a range of stakeholders.

We will focus on the following two research questions—which were taken-up (implicitly or explicitly) in the organizing of the SUBCOP project:

1. How do researchers/developers, in the context of a research and innovation project, without expertise in VSD, learn about end-users’ perspectives and values during the innovation process?
2. How do researchers/developers, in the context of a research and innovation project, without expertise in VSD, take into account these values, and related design criteria, in their decision-making during the research and innovation process?

We conducted case studies of five development processes ‘from within’. We did that as members of the Ethics Advisory Board (EAB). We spoke with two tool developers of each of these processes about the above-mentioned two questions five times (before every SUBCOP biannual consortium meeting) by phone-, skype- or face-to-face-meetings of 1 h each. Notes of these interviews were made and the notes were approved by the interviewees. These interviews were semi-structured; the following agenda was used as a guideline for the interviews:

- The tool developers gave a brief update on the development process so far and on the current status of the tool developing;
- We, as the EAB, asked several questions about the ways in which the tool developers (plan to) take into account different stakeholders’ views and values;
- Then a joint discussion about several specific (ethical) questions or issues regarding the tool which was developed.

The objective of the EAB was to make the researchers and developers more aware of the ethics at play in the SUBCOP project, and whether they encountered ethical issue with regards to the tool they were developing. As a side effect of these dialogues, we were also able to gain an understanding of the research and development processes. During these dialogues, we found that the researchers and developers were mainly concerned with end-users, as key stakeholders, i.e., the first responders, who are directly involved in the incident, and also with the bystanders, i.e., the people who are present at the incident, and the suspect (who may prove to be innocent).

### Values

Value Sensitive Design provides no methodological account for distinguishing (genuine) moral values from (mere) preferences, wishes, and whims of those involved in the design process. Mander-Huits (2011) and Iverson et al. (2010), however, discussed a variety of methods for discovering the main moral values in collaboration with stakeholders. In this SUBCOP-project we chose Participatory Design workshops (see Halloran et al. 2009) which are referred to in this project as End-user Meetings.

The objective of the first two End-user Meetings was to identify the main values, the problems faced by end-users in a suicide bombing incident, and establishing a scenario. One way to identify the main values is to establish a set of ‘core values’ that all relevant stakeholders sign-up to. Additionally, an ‘ethics checklist’ can act as an aide memoire and can be modified to apply specifically to the project at hand. Application of the checklist can highlight misunderstandings of terminology and conceptual problems associated with the rationales that lie behind conventional ethical principles. A basis for core values can be found in Van de Poel (2015). Van de Poel defined a set of main values, in which he distinguished between instrumental values (effectiveness, efficiency, reliability, robustness, maintainability, compatibility, and quality) and final values (safety, health, human well-being, and sustainability). In the SUBCOP project, three main moral values were identified according to an ethics checklist. The tools developed in this process would focus on these three values:

- Effectiveness: Ability to remove the threat (i.e. the PBIED suspect)
- Safety: Ability to survive the threat (i.e. by-standers, first responders, PBIED suspect)
- Utility (or efficiency): Ability to properly utilize the tool (i.e. first responders) of the different tool choices (softer methods/containment/NLW) when interfering with a PBIED suspect.

These three values were used to select the design criteria. To elicit the required information for these design criteria a semi-structured interview process was designed, using a questionnaire format based upon the history of PBIED’s in the country of the interviewees, i.e., the end-users. The questions for the end-users were divided into two: firstly for those countries that had had previous experience of “History of ‘In Country’ Suicide Attacks” (total of 34 questions), and secondly for those which had “No Known Previous History of ‘In Country’ Suicide Attacks” (23 questions). The questions were divided into the following sections:

- Problem identification (e.g., “Have you or your organisation ever arrested persons suspected of involvement in planning a terrorist attack? If yes, what was the incident?”)
- Terrorist and Terrorist Device Characteristics (e.g., “have you or your organisation undertaken research into the characteristics of suicide terrorist attacks? If yes, what were the outcomes?”)
- Tactics, Training and Procedure (e.g., “Can you describe the Command structure you have for managing these types of incident?”)
- Equipment (e.g., “What type of equipment do your response forces use during these types of incident?”)

To ensure that the responses received are in accordance with expectations, the questionnaires were piloted with two end-users. The final list of participants consisted of thirteen agencies in the end-user questionnaire which was representative of the wider EU end user community. On the basis of the results from the questionnaires, some striking issues were selected and explored in more detail during the interviews. The data from the questionnaires and the interviews were processed using a simplified variant of Ground Theory (see, e.g., Thornberg and Charmaz 2012) to identify key emerging issues, a Basic data table, and simple comparative analysis. From the questionnaires and the interviews the constraints faced by the end-users, and a list of end-users desirable technologies emerged. In a further meeting in the 12th month of the project with the end-users and all the participants of the SUBCOP project (including the tool developers) the design criteria were defined and the decision was made regarding the tools to be developed.

To weigh the different design criteria, and to deal with potential trade-offs, a multi-criteria analysis was used based on Bayesian networks (see, e.g., Delcroix et al. 2013). Multi-criteria analysis involves the selection of the best tools from a set of alternatives, each of which is evaluated against multiple, and often conflicting, design criteria. Most of the existing methods only focus on decisions under certainty. By basing the analysis on Bayesian networks, one can deal with uncertain interactions between design criteria and other factors, such as uncertainties in scenario descriptions and cultural aspects. Another strength of a Bayesian network is that different levels of detail can be taken together in one model, e.g., constructive/causative relations between variables can be defined qualitative as well as quantitative, and can be used within one model.

Since the priority ranking of the design criteria depends on situational factors, a scenario was established: an underground station based on the discussions in the first two End-user Meetings. This scenario was developed in order to clarify the context of use of the various tools, and helped to focus on the development of these tools (if the context of the use is unclear, it is hard to direct the tools’ development). Although this scenario was already mentioned in the project plan, it was only after several months (after the first two End-user Meetings), that the project team members and end-users had sufficient consensus, commitment and clarity on this. A scenario has a huge impact on the determination of the design criteria, since it can place constraints on the practicalities and deploying tools. Eventually, five tools were selected to investigate and seemed promising to be used in the chosen scenario (see the next section) in terms of the three main values: effectiveness, safety, and utility. According to the project plan, the research and development efforts for these tools did not start until the meeting with end-users in the 12th month of the project.

After this meeting, five more End-users meetings (workshops) were organised during the project. The objective of these meetings was for the end-users to have an input into the developing tools. In these meetings the tool developers presented their planned research and development outlines, and raised specific questions to the end-users. The outcomes of these discussions were summarized in the End User Advisory Board meeting minutes.

One of the researchers of this project articulated the added value of these End-user Meetings as follows: “The end-users added real world experience and guidance for tool development. Without that feedback we would have been designing to a speculative scenario or need”, which would have been like “designing in the dark”. Users’ feedback, he argued “greatly enhances the chances of the tool you develop being truly useful and subsequently deployed.”

For example, in the discussions with the end-users in one of the End-user Meetings it became clear for the researchers that a successful deployment of tasers differs from country to country, and that different rules of engagement for different contexts are necessary. E.g., in a country where end-users are less used to suicide bombing will be concerned, e.g., with not hurting a suspect-who-turns-out-to-be-innocent. In contrast, the people in a country, which has a history of suicide bombing, will be more concerned with by-standers’ wellbeing and less with suspects, especially if they turn out to be suicide bombers.

## Cases

In the SUBCOP project five selected tools were developed or studied, in five sub-projects, executed by different teams of researchers and developers of different organizations:

- An *Acoustic Warning Signal Projector* (A-WASP) (by Company A)
- *Electronic Counter Measures* to prevent remote detonation (by Research lab B)
- *Procedures for using Electroshock devices* (‘tasers’) (by Research lab B)
- A system that produces a *Water Mist*, for blast and fragmentation mitigation (by Research lab C)
- A *Protective Shield*, for blast and fragmentation mitigation (by Research lab C)

Some of these tools are primarily aimed at protecting bystanders, e.g., the Water Mist, or protecting first responders, e.g., the Protective Shield and Electronic Counter Measures. Electroshock devices are intended to approach/engage suspects, and, if needed, to incapacitate them. These are also tools to prevent detonation, together with the Electronic Counter Measures. A-WASP was developed to separate the bystanders from the suspect. See Table 1.
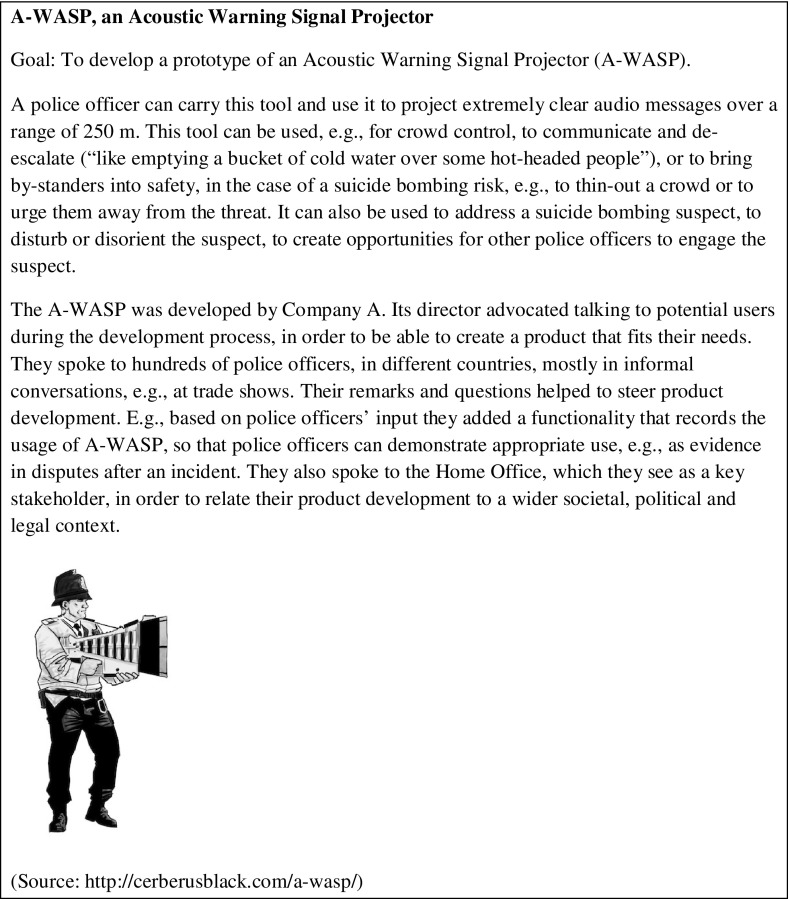
 
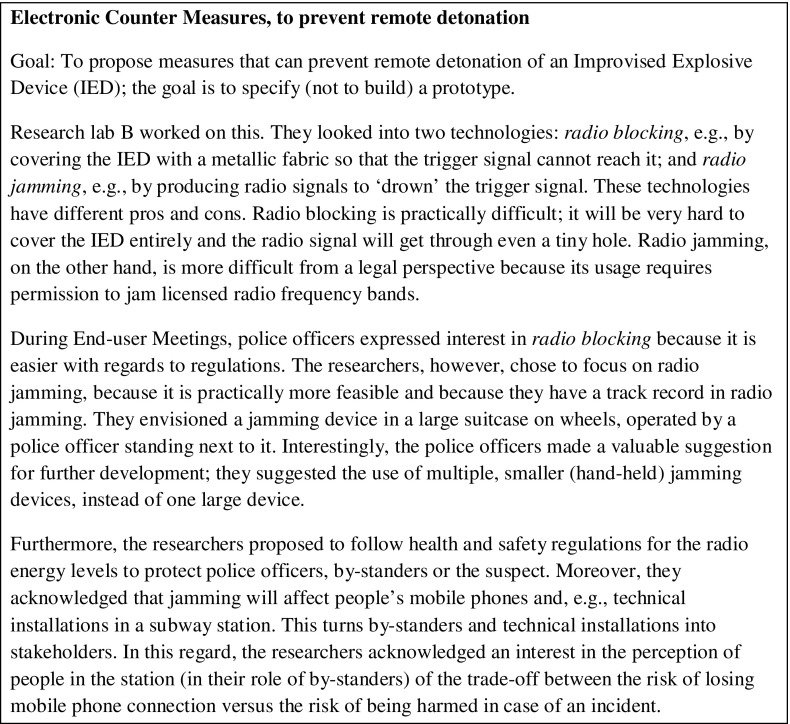
 
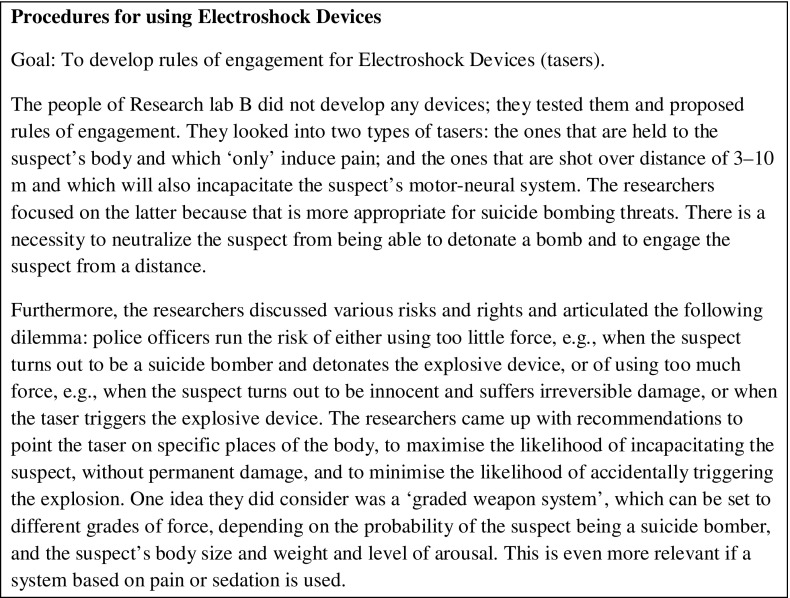
 
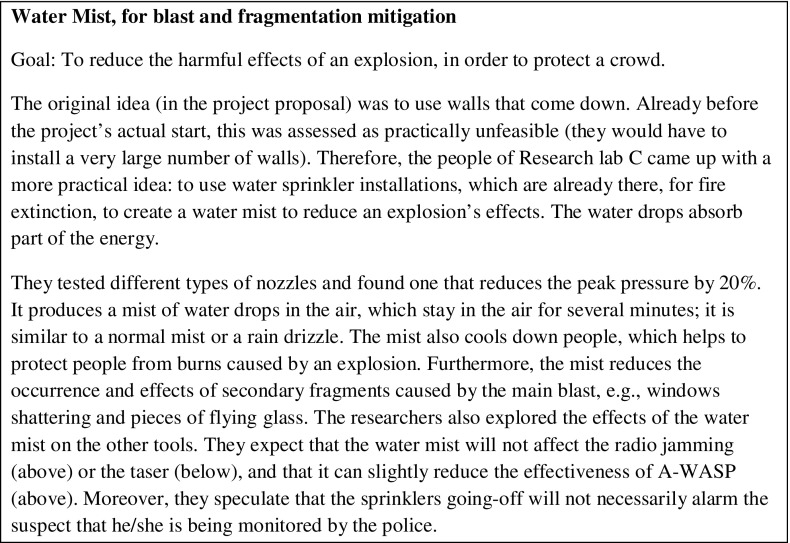
 
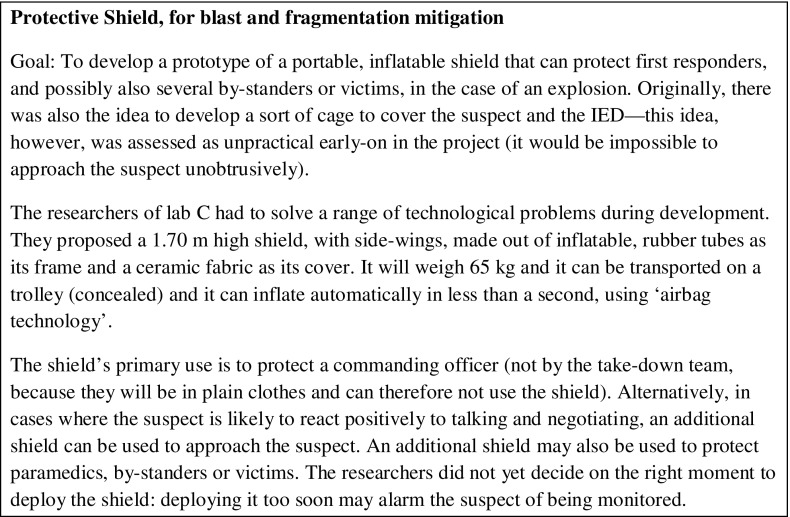

**Table 1:** The aims of the tools

Tool	Aim				
	Protect by-standers	Protect first responders	Interact with suspect	Prevent detonation	Reduce effects of explosion
A-WASP, an Acoustic Warning Signal Projector	X^+^		X^+^		
Electronic Counter Measures, to prevent detonation of an IED				X^+^	
Electroshock Devices, and procedures for using these			X^*+^	X	
Water Mist, for blast and fragmentation mitigation	X	X			X^0^
Protective Shield, for blast and fragmentation mitigation	X	X			X^0^

X*: for the Electroshock Device, ethical matters were discussed explicitly because their ‘tools’ are meant to engage suicide bomber suspects, and can potentially harm them

X^+^: for A-WASP, Electronic Counter Measures and Electroshock Devices, legal matters were discussed because their usage is related to all sorts of regulations for health and safety

X^0^: for the Water Mist and the Protective Shield, technological matters were discussed because ethical or legal matters were not (yet) urgent; these tools aim to protect people and cannot harm people

## Discussion

Let us now return to our two research questions—how researchers/developers learned about end-users’ perspectives and values, and how they took into account these values in their decision-making—and address them, based on the cases.

In the SUBCOP project the main activities to learn about end-users were the End-user Meetings. In these meetings key stakeholders were identified (first responders, by-standers, and suspect), main values were identified (effectiveness, safety, and utility), and a plausible scenario was established. Based on this information, the design criteria were defined. These criteria were used to select the most promising tools or procedures to develop or to study in more depth.

We can conclude that end-users did indeed participate actively and creatively: especially in taking into account their values and interests in decision making, such as the selecting of the most promising tools to develop or to study. Moreover, we can conclude that the researchers and developers, of Company A, and of Research labs B and C, took into account these values in their decision-making, and that they did this in different ways. We will discuss this below.

### Company A (A-WASP)

The researchers and developers of Company A that worked on the A-WASP were keen to combine technological innovation with commercial ambitions; they were keen to interact with end-users throughout the development process, to learn about their interests and needs by submitting a lot of questions to the end-users for assessment. The answers were systematically discussed with police officers, and responded systematically to what they learnt, and how the suggestions and ideas of the end-users would take into account in the design.

The principal researcher of Company A said about this process: “I have a background in photonics, and what often happens there, is: first developing a technology and then trying to find a problem to match that solution: technology push. When I created A-WASP, I wanted to do things differently. We spoke to as many police officers as possible, and approached the Home Office [the government department responsible for immigration, counter-terrorism, police, drugs policy, and related science and research^1^] and civil society organizations, such as the International Committee of the Red Cross (ICRC). We went to show them our very first early prototype. During all the sessions with our stakeholders we have expanded the A-WASP capability to include non-verbal sounds; easy access to banks of sounds; large on-board storage, evidence recording of all acoustic emissions (not just the siren noise), and multi-lingual character sets.” This enabled the developers to take into account the needs of different users. For example, the Welsh Police wanted to be able to make recordings of the device’s usage. They wanted to be able to demonstrate appropriate use: e.g., that the police officer did first warn the suspect and then decided to use force, rather than using force without warning first. So they added a feature that, once the device is turned on, makes recordings constantly, in buffering loops of 30 s (constantly deleting prior footage, to protect citizens’ privacy). This enables playback of 30 s of footage prior to the device’s usage, in order to provide context of its usage. Furthermore, the device’s usage by police officers will, of course, need to be compliant with existing working methods.

The researchers of A-WASP wanted to understand the potential users and their needs, and to develop tools for them which are effective and practical for them. They have clearly applied participatory design: the end-users were seen as co-designers in the design process, and the end-users could anticipate prototypes and future use. This is a shining example of how Value Sensitive Design could be applied. An explanation for this could be the commercial context of the company: its technological ambition to innovate is driven by the commercial ambition to serve users’ needs.

### Research Lab B (Electronic Counter Measures and Electroshock Devices)

The researchers and developers of Research Lab B worked on Electronic Counter Measures (ECM) and Electroshock Devices. With regard to the ECM, they were focused on developing basic radio jamming technology. During the End-user Meetings, it became clear that the end-users favoured radio blocking instead of radio jamming. Although the researchers and developers found this counter-intuitive, they did a thorough investigation of radio blocking and its effect, which have led to a better scientific explanation why radio jamming is a better option than radio blocking. Discussions with the end-users about how a threat situation would be handled today by first responders had led to an added functionality in the proposed design: small, man-carried ECM-devices for the first responders in close range were added to more powerful static ECM-equipment at a larger distance.

With regard to the electroshock devices, the researchers were well aware of differences in the needs of different police organizations in different countries. They acknowledged the different risks of using too little force (when the suspect turns out to be a suicide bomber and detonates the explosive device) or using too much force (when the suspect turns out to be innocent and suffers irreversible damage; or when the taser triggers the explosive device), and that these risks play out differently in different countries, e.g., the police in a country that suffers a lot from suicide bombing will prefer the risk of using too much force, whereas the police in a country that has very little experiences with suicide bombing will prefer the risk of using too little force.

Furthermore, in End-user meetings, the police officers had suggested to look into sedatives as a complement to the electroshock devices. The researchers, however, took little action on this suggestion since it is an ethically debatable method with a very high risk of lethal outcomes due to over-dosing. It is also questionable whether sedatives are allowed within the international humanitarian law, which forbids the use of chemical weapons. The researchers were focussed on their own interests in this technology, such as the degree of injury reversibility and effectiveness.

The researchers/developers of Research Lab B were keen to interact with end-users but they also had a relatively large amount of freedom to decide how to follow-up on their comments. End-users were seen as advisors and not as co-designers such as in the case of Company A. An explanation for this is that the work of the people of Research Lab B is mainly driven by studying physical phenomena and basic technological research, and that commercial goals, and associated commercial incentives to take end-users’ needs into account, are less prevalent.

### Research Lab C (Water Mist and Protective Shield)

The researchers and developers of Research Lab C worked on Water Mist and Protective Shield. They focused on applied technological research and development of proof-of-concepts. Moreover, their efforts were aimed at protecting people, not at interacting with, and potentially harming, people; therefore they believed that they did not have to worry a lot about ethical issues. As a consequence, they did not involve end-users in their innovation process. Even the decision to develop Water Mist instead of ‘walls that fall down in the case of incident’, which was originally planned, was based on their own ideas, and no end-user was involved in that decision. The only value of the End-user Meetings which they indicated was to get a response on acceptability and applicability of their ideas for developing tools for the chosen scenario.

The researchers and developers involved were not very keen to discuss ethics. They argued that this will be needed at the later stages of innovation, when commercial products are developed and used to assess, e.g., the police officers’ expectations with regard to protection and whether these expectations can be met.

In these cases, it is clear that the researchers and developers are not aware of how their design decisions influence the ethical impact of technologies, and that they are not very keen to proactively consider moral values throughout the process of technology design. The argument that the method of Value Sensitive Design is not applicable for basic technological research was disproved by Research Lab B. Also their research was mainly driven by basic technological research, but the input of end-users functioned as a reminder to look into alternative solutions (such as radio blocking and sedatives) and to justify design choices (e.g., the preference of end-users for radio blocking over radio jamming did prompt the researchers to further investigate radio blocking and to articulate why radio blocking is practically less feasible than radio jamming). Furthermore, the researchers and developers were confronted with conflicting values in their design: the protective shield will need to be small and light (for portability), and it will need to be large and strong (for protection). Involving the end-users as co-designers in the design process could help to deal with these conflicting values, where the researchers and developers learn the realities of the end-users’ situation while the end-users articulate their desired aims and learn the appropriate technological means to obtain them (Robertson and Simonsen 2012).

## Conclusions and Recommendations

We set-out our exploration with questions about how researchers and developers can learn about end-users’ values, and how they can take these values into account in their decision making in the innovation process. Based on an analysis of five research and development processes, we found that the organizing of End-user Meetings, in which researchers, developers and (potential) end-users collaborate, is an effective way to do this (to learn about end-users’ values and to take these into account in the innovation process). Moreover, we found the following general benefits of such an approach to end-user involvement (based on surveys amongst researchers and developers in these five cases, near the end of the project):

- End-user Meetings helped to identify and understand end-users’ main moral values (effectiveness, safety, and utility) with regard to counteracting or preventing suicide threats by participatory design workshops, i.e., the End-user Meetings in this project.
- These meetings also helped to established a commonly shared vision of the context of use (scenario) in which the tools would need to be useful and usable, i.e., a suicide bombing threat in a major public underground transport hub.
- Articulating this scenario helped to guide discussions of the practical acceptability and applicability of the five tools in various phases of development, e.g., to articulate technical requirements with respect to the values effectiveness, safety, and utility, and to test these, and also to regularly evaluate whether the researchers and developers were “on the right track”.

The familiar statement in the literature that many engineers are not aware of how their design decisions influence the ethical impact of technologies and even the ones who are aware have seldom received training on how to account values in design (e.g., Van den Hoven et al. 2015) can be confirmed when looking at the SUBCOP project. The people of Research lab C thought that they could do their research without end-users’ input since they believed that ethics are irrelevant in basic technological research. This was different for the people at Research lab B; they were keen to interact with the end-users but found it challenging to involve end-users in the design process and to take into account their input in the development process. A ‘text book’ example of Value Sensitive Design was the approach of Company A; they actively sought interactions with end-users, were curious about their needs and make significant efforts to take their input into account in the development process. They did this systematically, e.g., by using questionnaires to start the discussion, and by implementing different features to satisfy the needs of different types of end-users.

It can be expected of government sponsored university departments or research organizations that they engage in VSD (e.g., VSD pioneers Batya Friedman and Peter work in academia), or Participatory Design (PD) (Van der Velden and Mörtberg 2015) (e.g., pioneers of classical or ‘Scandinavian’ PD typically worked in academia), in order to take into account ethical or societal questions. However, based on our case study of Company A, we speculate—possibly somewhat counterintuitively—that a commercial context, with its focus on understanding and satisfying customers’, can also very well promote VSD or (‘contemporary’ or ‘international’) PD.

This finding can be useful for organizing and managing projects in commercial contexts and without involving experts of VSD. We speculate that researchers and developers can do something-like-VSD by ‘simply’ organizing a series of meetings with end-users, and listening to them, and organizing a series of critical reflections on ways to take into account the values expressed by end-users.

## Footnotes

1
https://www.gov.uk/government/organisations/home-office.
